# A 23‐year‐old woman with a headache and imbalance

**DOI:** 10.1111/bpa.70008

**Published:** 2025-03-30

**Authors:** Lukas Marcelis, Andrew Folpe, Sounak Gupta, Cinthya J Zepeda Mendoza

**Affiliations:** ^1^ Department of Pathology University Hospitals Leuven Leuven Belgium; ^2^ Department of Laboratory Medicine and Pathology Mayo Clinic Rochester Minnesota USA

**Keywords:** *DICER1*, intracranial sarcoma

BOX 1 **Virtual glass slide**

Access at https://isn‐slidearchive.org/?col=ISN&fol=Archive&file=BPA‐25‐02‐CIR‐059.svs.

## 1 CLINICAL HISTORY AND IMAGING

**FIGURE 1:**
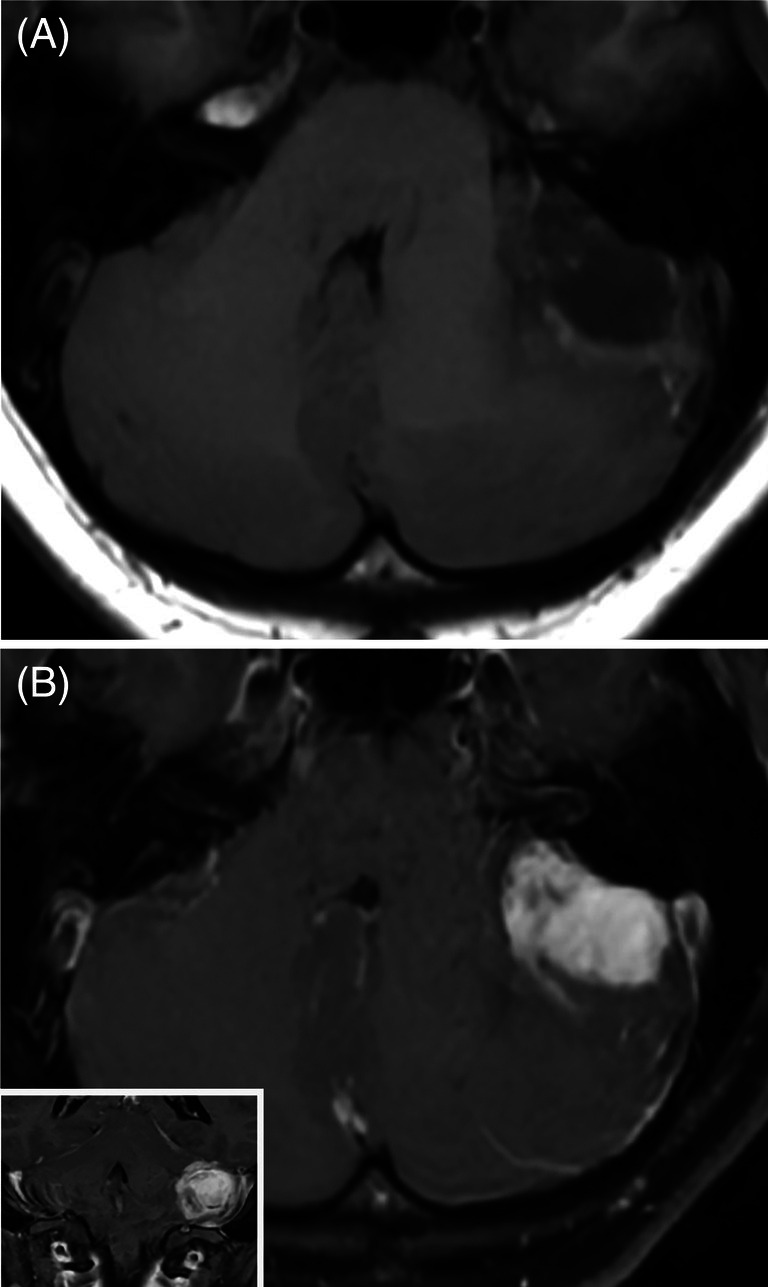
MRI (T1‐FLAIR) pre‐contrast (A) and post‐contrast (B) showed a heterogeneously enhancing mass in the left cerebellar hemisphere.

A 23‐year‐old woman presented to the emergency department with headaches and imbalance. Computer tomography imaging was obtained first and showed the presence of a hemorrhagic lesion in the left cerebellum (not shown). Subsequent magnetic resonance imaging showed a heterogeneously enhancing mass in the left cerebellum measuring 3.4 × 2.1 × 2.4 cm with peripheral blood products (Figure 1). There was associated vasogenic edema in the left cerebellum with mass effect on the posterior lateral brainstem and encroachment upon the 4th ventricle without hydrocephalus. An operative resection of the lesion was then pursued.

## 2 FINDINGS

**FIGURE 2:**
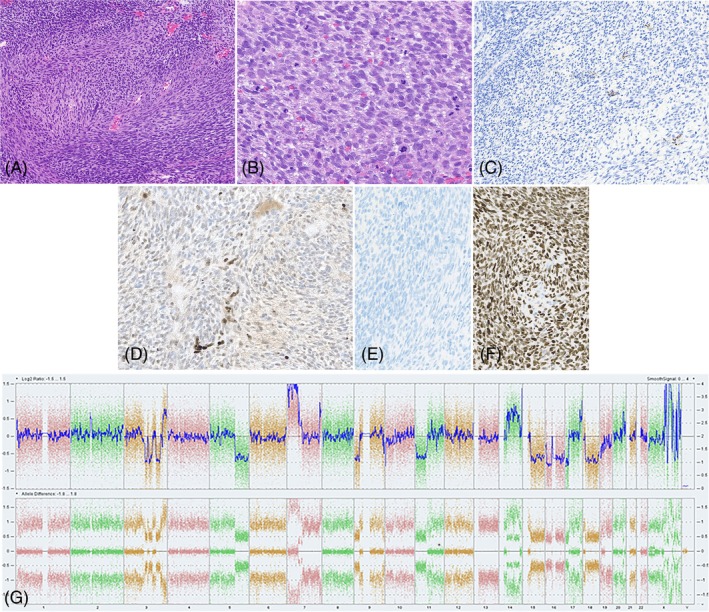
Morphologic, immunohistochemical, and molecular features of cerebellar mass (A 100X, B 400X, C 250x, D 320X and E + F 200X). (A, B) H&E‐stain showed a compact cellular lesion composed of monomorphic spindle cells with partial fascicular growth pattern and abundant mitoses; (C) desmin‐stain is negative in the neoplastic cells with positive internal control (vessel walls); (D) H3K27me3‐stain showed loss of nuclear staining in the neoplastic cells with retained nuclear staining in endothelium; (E) SSX‐SS18‐stain is negative and (F) SSX‐C‐stain is diffusely positive. (G) Microarray copy‐number analysis revealed a complex unbalanced genome with heterozygous deletion of 9p (including *CDKN2A/B*) and Xq chromothripsis, among other abnormalities.

H&E‐stained section revealed a highly cellular spindle cell neoplasm with a prominently fascicular growth pattern and no readily identifiable stroma (Figure 2A and Box 1). The tumor had very high mitotic activity (up to 10 mitotic figures in a single high‐powered field) (Figure 2B). The spindle cells were monomorphic with oval to elongated nuclei and inapparent nucleoli (Figure 2B).

The immunophenotype of the tumor did not reveal a clear line of origin, with negative staining for GFAP, OLIG2, S100 protein, SOX10, desmin (Figure 2C), pankeratin, synaptophysin, and only focally positive vimentin. Delicate pericellular reticulin deposition was noted, supporting the hypothesis of a sarcomatous neoplasm. The proliferation index (Ki‐67) was over 80% in accordance with the high mitotic activity. Subsequent staining for H3K27me3 showed loss of nuclear expression (Figure 2D); beta‐catenin staining was exclusively cytoplasmic, and SS18‐SSX fusion antibody staining was negative (Figure 2E), but SSX‐C terminus antibody staining was strongly and diffusely positive (Figure 2F).

DNA next‐generation sequencing (NGS) mutation analysis with a neuro‐oncology gene panel was performed and showed three clinically relevant mutations, one in *TP53* (c.493C > T (Exon 5)) and two mutations in *DICER1* (c.4405_4406del (Exon 23) and c.5425G > A (Exon 25)). RNA NGS gene fusion analysis with a sarcoma gene panel did not reveal any gene fusions. Chromosome microarray (CMA) analysis was performed (Applied Biosystems (Affymetrix) OncoScan) and was consistent with a clonal neoplastic process with chromosomal complexity including heterozygous deletion of 9p (encompassing *CDKN2A/B*) and chromosome Xq chromothripsis, among others (Figure 2G).

## 3 FINAL DIAGNOSIS

Primary intracranial sarcoma, DICER1‐mutant.

## 4 DISCUSSION

This was a difficult to diagnose as a high‐grade spindle cell neoplasm without specific morphological or immunohistochemical features. The initial differential diagnosis based on imaging included medulloblastoma given the anatomical location and the patient's age. The morphologic presentation of a sarcomatous, monomorphic spindle cell proliferation raised a broad differential diagnosis including malignant peripheral nerve sheath tumor (MPNST), primary intracranial sarcoma, DICER1‐mutant, and synovial sarcoma (SS). Subsequent targeted immunohistochemical stains showed loss of nuclear H3K27me3 staining as can be seen in MPNST, negative staining for the SSX‐SS18 fusion protein, but positive staining for the SSX‐C protein. While the SSX‐SS18 antibody is a gene fusion site‐specific antibody that is highly specific for SS, the SSX‐C antibody targets the C‐terminus of the SSX protein and can also be positive in a subset of histological mimics of SS including MPNST [1]. Loss of nuclear H3K27me3 is reported in both MPNST and primary intracranial sarcoma, DICER1‐mutant [2], while to the best of our knowledge, no data on SSX‐C staining in the latter entity is currently available. While Xq chromothripsis was present in this tumor, no apparent copy‐number changes were found at Xp where *SSX1* is located. RNA NGS with a sarcoma gene panel including *SS18* did not show any gene fusions.

DNA NGS revealed mutations in *DICER1* exon 23 and 25. Exon 25 encodes the RNase IIIb domain and is a highly recurrent mutation hotspot in primary intracranial sarcoma, DICER1‐mutant [3]. This entity was first described by Koelche et al. in 2018 [3] and, because of its rarity, it is difficult to determine the prognosis, but an aggressive clinical course is suspected. The case presented here is challenging as it lacks more specific histological features such as cartilaginous or myogenic differentiation, myxoid stroma, and eosinophilic cytoplasmic globules. Most cases are also supratentorial, while this lesion was in the cerebellum, although the dural involvement is typical for primary intracranial sarcoma, DICER1‐mutant.

## AUTHOR CONTRIBUTIONS

Lukas Marcelis wrote the manuscript and participated in diagnostic discussions. Dr. Andrew Folpe participated in making the diagnosis as a soft tissue tumor expert and reviewed and edited the draft. Dr. Sounak Gupta and Dr. Cinthya Zepeda Mendoza interpreted the molecular results, participated in making Figure 2, and reviewed and edited the draft.

## CONFLICT OF INTEREST STATEMENT

The authors declare no conflicts of interest.

## Data Availability

Data sharing not applicable to this article as no datasets were generated or analysed during the current study.
